# Plasma exosomal miR-142-3p induced by acupuncture protects against heart injury in rats with myocardial infarction by targeting Cofilin 2

**DOI:** 10.22038/ijbms.2025.84835.18361

**Published:** 2025

**Authors:** Yue Zhao, Ying Wang, Chunrong Guo, Qiyu Sun, Jinxia Mi, Pingping Lu, Shuijin Shao, Rong Lu, Haidong Guo, Qiangli Wang

**Affiliations:** 1 Department of Histoembryology, School of Integrative Medicine, Shanghai University of Traditional Chinese Medicine, Shanghai 201203, China; 2 Department of Chemistry, School of Pharmacy, Shanghai University of Traditional Chinese Medicine, Shanghai 201203, China; 3 School of Integrative Medicine, Shanghai University of Traditional Chinese Medicine, Shanghai 201203, China; 4 Research Center for Health and Nutrition, Shanghai University of Traditional Chinese Medicine, Shanghai 201203, China; 5 Department of Anatomy, School of Integrative Medicine, Shanghai University of Traditional Chinese Medicine, Shanghai 201203, China; 6 School of Traditional Chinese Medicine, Shanghai University of Traditional Chinese Medicine, Shanghai 201203, China

**Keywords:** Acupuncture, Cofilin 2, Exosomes, MicroRNAs, Myocardial infarction

## Abstract

**Objective(s):**

The protective effects of acupuncture on myocardial injury have been identified in clinical trials. However, there is still a lack of comprehensive understanding of its fundamental mechanism. This research aimed to clarify the roles of plasma exosomes in the cardioprotection of acupuncture.

**Materials and Methods:**

Myocardial infarcted rats were divided into control group, acupuncture group, and acupuncture plus GW4869 group. The rats without the performance of a myocardial infarction were divided into a sham group. Acupuncture was performed at bilateral PC6.

**Results:**

The results showed that the effects of acupuncture on increasing the thickness of the left ventricular infarct wall and inhibiting apoptosis of the damaged heart tissue were significantly reversed by GW4869. Among the five miRNAs of plasma exosomes increased by acupuncture, miR-142-3p was the unique miRNA up-regulated in myocardial tissue. Overexpressing miR-142-3p retarded oxidative damage of H9c2 by anti-apoptosis. miR-142-3p directly bound to and suppressed the expression of Cofilin 2 (CFL2). *In vivo* CFL2 expression was down-regulated by acupuncture and up-regulated by GW4869.

**Conclusion:**

Our results suggest that plasma exosomes transfer cardio-protective signals of acupuncture to the injured heart and confer cardioprotective effects, and miR-142-3p emerges as a prominent exosomal miRNA in the inhibition of myocardial apoptosis by targeting CFL2.

## Introduction

Cardiovascular disease (CVD) accounts for 26.8% of all deaths globally in 2021 (1). As a major manifestation of coronary artery disease, myocardial infarction (MI) caused over 3 million deaths in the United States between 1999 and 2020 (2, 3). In China, the overall MI mortality rate increased continuously from 2002 to 2020, with a rapid upward trend observed since 2005 (4). Severe MI kills nearly a billion cardiomyocytes due to prolonged ischemia, which leads to heart failure (HF) and even sudden cardiac death (5). Inhibiting cardiomyocyte death has been proposed as one of the therapeutic strategies for protecting MI survivors against HF (6). However, many randomized clinical trials have demonstrated that conditioning or cardioprotective interventions exhibit equivocal clinical effects on infarct size and patient clinical outcomes (7). Alternative therapies for transitory treatments with cardioprotective agents have been investigated by an increasing number of clinicians and pharmaceutical companies. Acupuncture has been utilized as a nonpharmacologic treatment for more than 2000 years, and its efficacy on myocardial ischemia and heart failure has been confirmed by many randomized clinical trials and animal experiments (8-10). The bilateral Neiguan (PC6), which is frequently utilized in Chinese medicine to address cardiovascular ailments, has demonstrated its efficacy in protecting the ischemic heart through multiple pathways, such as activating the adenosine receptor, alleviating mitochondrial damage, and attenuating autophagy in the ischemic heart (11-13). However, the mechanism by which Neiguan (PC6) triggers remote cardioprotection is still unclear. 

Exosomes are nanoscale vesicles composed of lipid bilayers and typically range in size from 30 nm to 100 nm, capable of being secreted by various cell types. Recently, the critical roles of exosomes have been uncovered in ferrying various signaling molecules, such as proteins, mRNAs, and miRNAs, to mediate communication between cells or even organs (14). Several studies have demonstrated that the cardioprotective effect of exosomes can be achieved by delivering miRNAs. For instance, exosomes induced by transient hindlimb ischemia can transfer miR-24, which opposes apoptosis, to provide cardioprotection in a rat acute ischemia/reperfusion (I/R) model (15). Plasma exosomes derived from individuals who engaged in long-term exercise exhibited cardioprotective effects by transferring miR-342-5p to alleviate I/R injury (16). The findings suggest that the cardiovascular benefits derived from remote acupuncture may be attributed to the mechanical stimulation of Neiguan (PC6), which triggers the release of exosomes from limbal tissues into circulation.

Numerous studies have explored the potential connections between acupuncture and exosomes. For example, Chen *et al*. demonstrated that serum exosomes induced by electroacupuncture exerted significantly protective effects against lipopolysaccharide-induced cardiomyopathy by delivering miR-381 (17). Xu *et al*. demonstrated that electroacupuncture up-regulated miR-210 expression in plasma exosomes within a middle cerebral artery occlusion model (18). Mechanistically, these exosomes promoted angiogenesis under hypoxic conditions via the HIF-1α/VEGF/Notch1 signaling pathway.

In this study, the involvement of exosomes in the cardioprotective effects of acupuncture at Neiguan (PC6) was initially established using a rat model of MI. On this basis, changes in miRNAs in the tissue of Neiguan (PC6) after acupuncture were detected by small RNA sequencing, and then the expression of these miRNAs was examined in plasma exosomes and infarcted myocardial tissue. Finally, exosomal miR-142-3p induced by acupuncture was identified as a novel cardioprotective molecule by down-regulating the expression of Cofilin 2 (CFL2). 

## Materials and Methods

### Reagents

Antibodies against cleaved caspase 3 (9664s Lot 22), BAX (2772s Lot 12) were obtained from Cell Signaling Technology, Inc. (Danvers, MA, USA). Antibodies targeting bcl-2 (26593-1-AP Lot 00113524), Cofilin 2 (11848-1-AP Lot 00021078), and caspase 3 (19677-1-AP Lot 00103232) were purchased from Proteintech Group (China). GW4869 (20418-50mg Lot CSN20418-003) was bought from CSNpharm (China). Polyethylene glycol (101913398 Lot BCBV7000) with Mn of 6000 was acquired from Sigma-Aldrich Co., LLC. (USA).

### Rat care and AMI surgery

All animal experiments conducted in this study were approved by the Institutional Animal Care and Use Committee at Shanghai University of Traditional Chinese Medicine (Approval No.: PZSHUTCM2401170002). The male Sprague-Dawley (SD) rats and Balb/c mice, aged eight weeks, were sourced from Slaccas Laboratory Animal Corporation in Shanghai, China. They were housed in a specific-pathogen-free facility at the animal experiment center of SHUTCM and provided with ad libitum access to food and water prior to their utilization. After a one-week acclimation period, the rats or mice were administered isoflurane anesthesia at a concentration of 5% for induction and 2% for maintenance. Subsequently, they underwent endotracheal intubation and were connected to the rodent ventilator (model UGO BASILE 7025, Milan, Italy). After confirming the absence of any signs of discomfort or pain, a thoracotomy was performed to access the chest cavity in rats or mice, specifically between the fourth and fifth intercostal spaces on the left side. Subsequently, a dilator was utilized to achieve optimal visualization of the heart, following which ligation of the left anterior descending coronary artery (LAD) was conducted 2 mm below the left auricle to induce AMI, employing previously established methodologies (19). The appearance of the left anterior myocardium, which converted from rosiness to paleness, indicated the successful establishment of an AMI model. The same procedures were performed in the sham-operated animals except for LAD ligation. 

### Acupuncture intervention

Neiguan (PC6) was located at the distal 1/6 point on the imaginary midline connecting the palm crease and cubital crease of the inner forelimb (20). On the second day after AMI, the rats were divided into three groups: sham, control, and Acu (with six rats in each group), using a randomization method. All the animals were individually confined in special cylindrical devices with their limbs extended. For the Acu group rats, the sterilized Huatuo needles (0.25 mm×13 mm; Suzhou Medical Appliance Manufactory, Jiangsu, China) were perpendicularly inserted in bilateral PC6 at a depth ranging from 2 to 3 mm and manually rotated back and forth *in situ* for 5 min, with a frequency of 120 times/min (21). For the control group, similar operations were performed at the tail non-acupoint of the rats. The sham-operated animals were only restrained without acupuncture intervention. Acupuncture or sham treatment was performed once daily for 6 days. 

### GW4869 treatment in the acupuncture-intervened rats

GW4869 was used to inhibit exosome secretion to clarify the roles of exosomes following the effects of acupuncture. It was dissolved in DMSO (0.005%). On the second day after AMI, the rats were divided into four groups: sham, control, Acu, and Acu plus GW4869 (with six rats in each group), employing a randomization method. The first three groups were treated as above. The rats in the Acu plus GW4869 group were administered a single intraperitoneal injection of GW4869 at a dose of 1.5 mg/kg (22), one hour prior to daily acupuncture. 

### Histological examination

Seven days after AMI, all the animals were anesthetized with isoflurane as described above. The rats were subjected to transcardial perfusion with 0.1 M PBS, followed by fixation with a 4% paraformaldehyde solution. The hearts were then cut transversely at the widest parts of the infarcted regions. The tissues were immersed in Tissue-Tek OCT compound (Sakura, Tokyo, Japan), promptly frozen in liquid nitrogen, and stored at -80 ^°^C. The blocks were pre-placed in the freezing microtome (Thermo Scientific, USA) to ensure temperature stabilization, and then serially sliced into 5-µm sections at -20 ^°^C. Ten consecutive sections from each block underwent Masson’s trichrome (Masson) staining. The images were obtained utilizing a light microscope manufactured by Olympus in Japan. The assessment of infarct size and left ventricular wall thickness was performed according to established protocols (19).

### In situ identification of apoptotic myocytes

Seven days after AMI, the Terminal deoxynucleotidyl transferase (TdT)-mediated dUTP nick-end labeling (TUNEL) assay was utilized to identify apoptotic cells in the infarcted hearts, following the protocols provided by Beyotime (Shanghai, China). The frozen sections, which were prepared as above, underwent three rounds of PBS washing, followed by a 5-minute incubation at room temperature with 0.5% TritonX-100. Subsequently, the specimens were exposed to the TUNEL reaction mixture containing TdT and FITC-dUTP for 60 min in a dark, humid environment (23). 

Following nuclear staining with DAPI, the results of the TUNEL assay were visualized using a fluorescence microscope (model IX50) manufactured by Olympus in Japan.

### Western blot analysis

Seven days after AMI, rats were anesthetized with isoflurane as described above, and the hearts were quickly removed. The left ventricular anterior wall of hearts was promptly frozen with liquid nitrogen and maintained at a temperature of -80 ^°^C for subsequent examinations. For western blot analysis(24), the samples of tissues and cells were homogenized in ice-cold RIPA lysis buffer, supplemented with a combination of protease inhibitors and phosphatase inhibitors. The tissue lysates were subjected to centrifugation at 12,000×g for 20 min at a temperature of 4 ^°^C. After performing BCA analysis (Beyotime, Shanghai, China) to determine the protein concentration in the supernatants, 40 μg of proteins were separated on a 12% SDS-polyacrylamide gel and subsequently transferred onto an NC membrane (Merck, Darmstadt, Germany). The primary antibodies, including anti-cleaved caspase 3 (CC3) antibody (1:1000), anti-caspase 3 antibody (1:3000), anti-Bcl-2 antibody (1:1000), anti-BAX antibody (1:1000) and anti-Cofilin 2 antibody (1:1000), were applied to the membrane and incubated overnight at a temperature of 4 ^°^C. Subsequently, the secondary antibodies conjugated with peroxidase were incubated with the membrane for one hour at room temperature. The bands on the membrane were subsequently detected using enhanced chemiluminescence western blot detection reagents (NCM Biotech, Suzhou, China) for visualization. Meanwhile, all images were obtained by using a Molecular Imager system (Bio-Rad, California, USA). The relative value of the band density was analyzed by ImageJ software.

### MiRNA sequencing and analysis of PC6 acupoint

The rats were sacrificed by euthanasia on the seventh day after MI to clarify the changes in the miRNA expression of PC6 acupoint. The PC6 tissues, including skin, subcutaneous tissue, and muscle tissue, were harvested and stored in a -80 ^°^C ultra-low temperature freezer after snap-freezing in liquid nitrogen. A total of nine samples from the sham, control, and Acu group were promptly cryopreserved in liquid nitrogen and transported to Shanghai Majorbio Bio-pharm Technology Co., Ltd. for miRNA sequencing. Total RNA was extracted from the samples by using TRIzol^®^ Reagent (Majorbio, Shanghai, China) following the manufacturer’s guidelines. The RNA quality was then assessed using the 5300 Bioanalyser (Agilent, USA) and quantified with the ND-2000 (NanoDrop Technologies). Only high-quality RNA samples meeting the following criteria were utilized for library construction: OD260/280=1.8-2.0, OD260/230 ≥ 2.0), RNA Integrity Number (RIN) ≥6.5), 28S:18S ≥ 1.0, and concentration of ≥2 μg. A small RNA-seq transcriptome library was generated according to the manufacturer’s instructions, utilizing the QIAseq miRNA Library Kit (Qiagen, Hilden, Germany) and employing 1 μg of total RNA per sample. Subsequent sequencing was conducted on an Illumina NovaSeq X plus platform. The bioinformatics data were analyzed using the cloud-based platform provided by Majorbio Bio-Pharm Technology Co., Ltd (25).

### Plasma exosome isolation and identification

Exosomes were isolated from rat plasma as described (26). Seven days after AMI, a blood sample was collected from the anesthetized rats’ abdominal aorta prior to their sacrifice and subsequently centrifuged for 30 min at 1000 g in a tube containing anticoagulant. The plasma was subsequently transferred to a fresh tube and subjected to centrifugation at 2000 g for 15 min at 4 ^°^C to remove cellular debris. Thereafter, it underwent further centrifugation at 10,000 g for 30 min at the same temperature to eliminate large microvesicles. The supernatant was supplemented with an equal volume of 16% polyethylene glycol (PEG) with a molecular weight of 6000 (Sigma, USA). After thorough mixing, the mixture was incubated undisturbed overnight at 4 ^°^C. The following day, the samples were subjected to centrifugation at 16,000 g for one hour at 4 ^°^C. The precipitation was collected in 1 ml of PBS and subjected to ultracentrifugation at 100,000 g for 70 min to remove any particles associated with the contaminated protein and PEG. The resulting pellet was resuspended in 50 μl of particle-free PBS (pH 7.4) and stored at -80 ^°^C for future utilization.

The ultrastructure of the acquired exosomes was examined utilizing the Tecnai G2 Spirit Biotwin transmission electron microscope (FEI, Hillsboro, USA). Both the size distribution and concentration of the 10 times diluted exosomes were determined using nanoparticle tracking analysis (NTA) with Zeta View Particle Metrix 110 (Particle Metrix, Meerbusch, Germany. The marker proteins of exosomes (CD9, CD63, CD81, and Alix) were detected by western blot. The quantity of exosomes was identified utilizing a BCA kit (YEASEN, Shanghai, China) for measurement of total protein. 

### Cell culture and exosome treatment

H9c2 cells (Cobioer, Nanjing, China, catalogue number: CBP60588, RRID: CVCL_0286), derived from embryonic rat cardiomyoblasts, were cultured in a 96-well plate at a seeding density of 1×10^4^ per well. The culture medium consisted of DMEM supplemented with 10% exosome-depleted fetal bovine serum (FBS), 100 U/ml penicillin, and 100 μg/ml streptomycin. Incubation was conducted at a temperature of 37 ^°^C in an environment containing 5% CO_2_ for a duration of 24 hr. Cells were then incubated with different concentrations of exosomes (10, 20, 40, and 80 μg/ml) isolated from the sham, control, and Acu groups for 24 hr. The viability of H9c2 cells was evaluated using the CCK-8 assay (YEASEN, Shanghai, China)(27).

### In vitro and in vivo fluorescence tracing of exosomes

For* in vitro* tracing of exosomes in H9c2 cells, red fluorescent dye PKH26 was used to label plasma exosomes, following the guidelines provided by the manufacturer (28). The H9c2 cells were exposed to PKH26-labeled exosomes for three hours. Subsequently, the cells underwent three rounds of PBS washing and were fixed using 4% paraformaldehyde for 10 min. Finally, the cells were washed twice again with PBS. The cellular internalization of exosomes was visualized using a fluorescence microscope (Olympus IX50, Japan). The *ex vivo *fluorescence tracking of exosomes was conducted by labeling plasma exosomes with DiI, following the manufacturer’s guidelines. Then, the mice from both the control and acupuncture groups received a tail intravenous injection of 200 μg of DiI-labeled exosomes, following the previously described protocol (29, 30). The IVIS® Lumina II *in vivo* imaging system (PerkinElmer, Thermo Fisher, USA) was employed to visualize the localization of exosomes in various organs four hours later.

### RT-PCR detection

The RNAiso Plus and RNAiso for Small RNA kit (Takara, Dalian, China) were used to extract total RNA from H9c2 cells and small RNA from exosomes and tissues stored in an ultra-low temperature freezer seven days after AMI, as described in a previous study (31). The Fermentas cDNA kit (Fermentas, USA) was used to transcribe mRNA into cDNA. The Mir-X miRNA First-Strand Synthesis Kit (Takara, Dalian, China) was employed for reverse transcription of first-strand cDNA. For the quantification of cDNA, real-time RT-PCR was performed using TB Green Advantage RT-PCR Premix (Takara, Dalian, China). The relative expression of each mRNA and miRNA to the level of β-actin and U6 snRNA was determined using the delta-delta Ct method. All specimens were analyzed in triplicate. The forward and reverse primers for the Cofilin 2 are as follows: Forward: 5′ TTGTGAAGTTGCTACCTCTGAATG3′; Reverse: 5′ TTAAAGGTGCACTTTCAGGAGCC 3′. The forward primers utilized for the miRNAs of interest are as follows: miR-21-5p 5′ ACGTTGTGTAGCTTAT CAGACTG 3′; miR-21-3p 5′ TGCGCCAACAGCAGTCGA TGGG 3′; miR-27-5p 5′ GCGGCGGAGGGCTTAGCTGCTTG 3′; miR-31-5p 5′ CGGCGGAGGCAAG ATGCTGGCA 3′; miR-142-5p 5′ GGCCCATAAAGTAGAA AGC 3′; miR-142-3p 5′ CTCCTGTAGTGTTTCCTAC 3′; miR-223-5p 5′ TCGC GTGTATTTGACAAG CTGAGTTG 3′; miR-223-3p 5′ GAAGTTCGTCCTGTC AGTTTGTC 3′. The reverse primer for miRNA was the mRQ 3′ Primer (Takara, Dalian, China).

### Cytoprotectivity of exosomes

The potential protective effects of exosomes against H_2_O_2_-induced oxidative stress were evaluated by pre-treating H9c2 cells with exosomes isolated from the sham, control, and Acu groups for 24 hr. After replacing the complete culture medium with a serum-free medium, the plate underwent a 2-hour incubation period with H_2_O_2_ at a concentration of 400 μM (32). The viability of H9c2 cells was evaluated utilizing the CCK-8 assay. To investigate the protein expression of H9c2 cells after H_2_O_2 _treatment, the cells were cultured in a six-well plate by seeding 5×10^5 ^cells per well, and similar experiments were performed as described above. 

### Prediction and identification of the target gene

The prediction of target genes for rat miR-142-3p was performed utilizing bioinformatics tools including miRanda, miRDB, and TargetScan(16). Among the possible miR-142-3p target genes, the focus of this research was primarily on CFL2, which plays a pivotal role in MI and the apoptotic pathway. A dual-luciferase reporter gene assay was performed in 293 T cells to determine the target gene. In short, the 3′ UTR and its site-specific mutated variants of CFL2 were cloned into the XbaI/XbaI site of the GV272 vector (GeneChem Biotechnology Company, Shanghai, China), which contained firefly luciferase reporter genes. Then, 293 T cells were cultured in a 24-well plate by seeding 1×10^5 ^cells per well. The plasmid constructs that have undergone recombination (CFL23′-UTR and CFL23′-UTR-mut, 0.1 μg) were separately transfected into the 293 T cells with Renilla luciferase internal control plasma (pRL-TK, 0.02 μg) and miR-142-3p (0.4 μg) or the negative control of mimic plasma (NC mimic, 0.4 μg) by X-tremegene HP DNA transfection reagents (1 μl, ROCHE, Basel, Switzerland) as follows: Luc-CFL2 3′-UTR-NC+miR-142-3p-NC, Luc-CFL2 3′-UTR-NC+miR-142-3p, Luc-CFL2 3′-UTR+miR-142-3p-NC, Luc-CFL2 3′-UTR+miR-142-3p, Luc-CFL2 3′-UTR-Mut+miR142-3p-NC, and Luc-CFL2 3′-UTR-Mut+miR142-3p. The dual-luciferase reporter assay system (Promega, Madison, USA) was employed to evaluate the activity of firefly luciferase and Renilla luciferase 48 hr post-transfection, following the manufacturer’s guidelines. The experimental treatments were replicated three times, with a minimum of three replicates per treatment.

### Lentivirus preparation and transfection

To further verify the inhibition of CFL2 expression by miR-142-3p and the effect of overexpressing miR-142-3p on cell apoptosis induced by H_2_O_2_, miRNA-142-3p mimic, miR-142-3p sponge, and scrambled control lentiviruses were designed and synthesized by GeneChem Biotechnology Company (Shanghai, China). The H9c2 cells were evenly distributed into 24-well plates at a concentration of 5×10^4^ cells per well. When the cells reached 70% confluence, lentiviral vectors were added to the culture medium at an MOI of 50, followed by 5 μg/ml polybrene. The miRNA-142-3p-overexpressing cells, miR-142-3p sponge-expressing cells, and scrambled control cells were selected using either puromycin or neomycin. The expression of CLF-2 in infected H9c2 cells was examined by RT-PCR, and the viability of H9c2 cells was evaluated after incubation with H_2_O_2 _using a CCK-8 assay as described above.

### Statistical analysis

Each experiment was independently repeated at least three times, and the mean±SEM was used to represent all derived statistical values. GraphPad Prism 6 (GraphPad, San Diego, CA, USA) software was used to determine the statistical significance between datasets. Student’s t-test was used for comparing two groups, while ANOVA, followed by Tukey’s multiple comparison test, was utilized for comparing multiple groups. Statistical significance was considered when the *P*-value was less than 0.05.

## Results

### Acupuncture at Neiguan (P6) attenuates heart injury after MI

**Figure 1:**
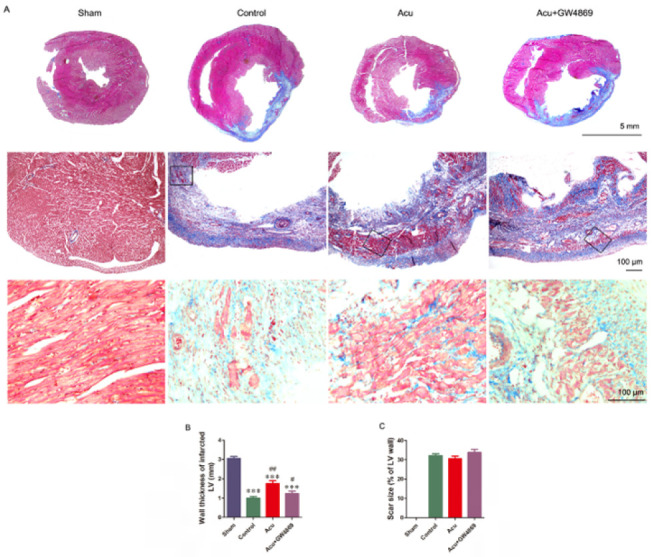
Acupuncture at Neiguan (P6) thickened the infarcted left ventricular wall and inhibited apoptosis of the infarcted heart in rats

The infarcted hearts were subjected to Masson’s trichrome staining to examine the histological changes. Seven days after MI, a decrease in the left ventricular wall thickness was apparent in the control group (0.90±0.12) compared with the sham group (2.34±0.16). Acupuncture increased the left ventricular wall thickness (1.69±0.19), where more cardiocytes survived compared with the control group (Figures 1A and B). However, the size of the infarct showed no discernible disparity between the control and Acu groups (Figure 1C). The expression levels of caspase 3 and CC3 were evaluated by western blot to investigate apoptosis of the infarcted heart. The results revealed an up-regulation of both caspase 3 and CC3 in the control group, while a down-regulation was observed in the Acu group (Figures 1D-F). This data indicated that acupuncture at Neiguan (P6) could improve the survival of cardiac cells after MI through anti-apoptosis.

### Cardioprotective impact of acupuncture is attenuated by the inhibition of exosome release

**Figure 2:**
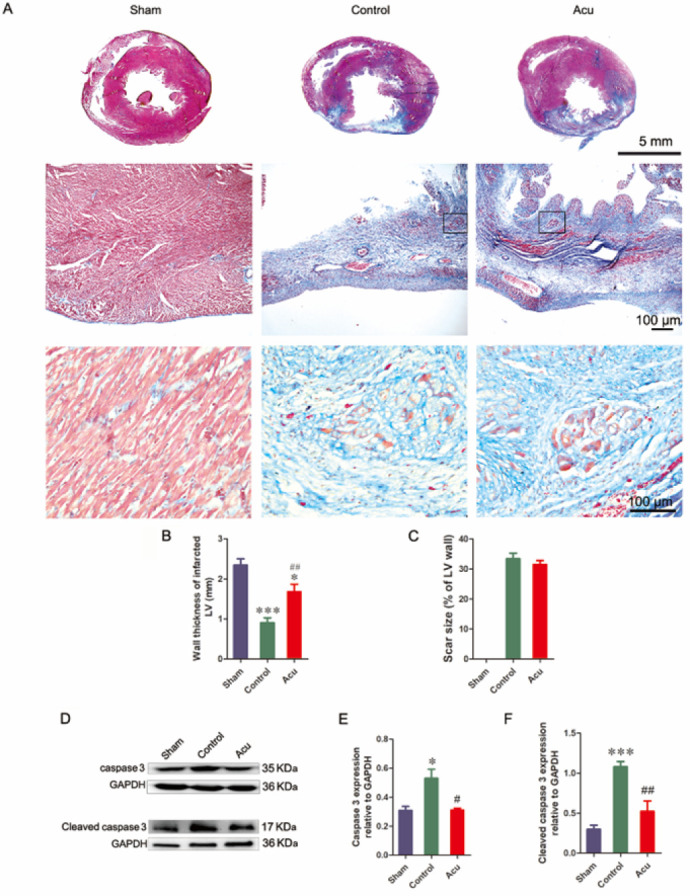
GW4869 weakened the cardioprotective effects of acupuncture in rats

**Figure 3:**
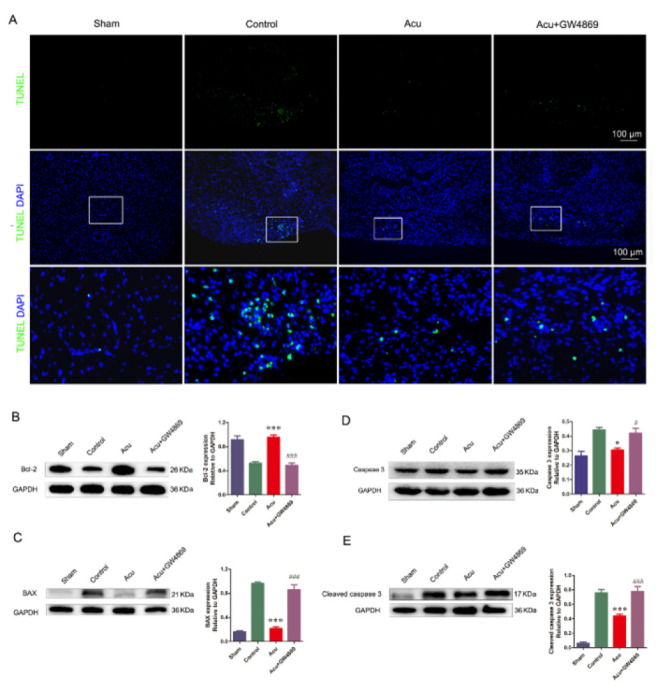
Anti-apoptosis effect of acupuncture on an infarcted heart was dampened by GW4869 in rats

For clarification of the role of exosomes in acupunctural cardioprotection, their secretion was blocked by intraperitoneal injection of GW4869 (1.5 mg/kg, qd) one hour before daily acupuncture. After a week following MI, Masson’s trichrome staining showed that the thickness of the left ventricular wall in the Acu plus GW4869 group obviously decreased (1.23±0.13) compared with that in the Acu group (1.75±0.15). The surviving cardiocytes in the infarcted region were fewer in the Acu plus GW4869 group compared to the Acu group (Figures 2A-B). Similar to the above results, no difference in the infarct size was observed in the control, Acu, and Acu plus GW4869 groups (Figure 2C). The TUNEL staining results revealed a significantly higher presence of apoptotic cells in the infarcted area of the heart within the control group, whereas a comparatively lower number of apoptotic cells were observed in the Acu group. In addition, apoptotic cells were more evident in the Acu plus GW4869 group than in the Acu group (Figure 3A). The apoptosis-associated protein was assessed via western blot analysis. The results revealed that the Acu group exhibited up-regulated expression of Bcl-2 and down-regulated expression of BAX, caspase 3, and CC3 compared to the control group. Importantly, GW4869 injection effectively restored the altered expression levels of these proteins induced by acupuncture treatment. (Figures 3B-E). Collectively, this data indicated that inhibition of exosome generation with GW4869 diminished acupuncture-induced myocardial protection.

### Plasma exosomes from acupuncture accumulate in the infarcted heart and ameliorate cardiocyte injury induced by H2O2

**Figure 4:**
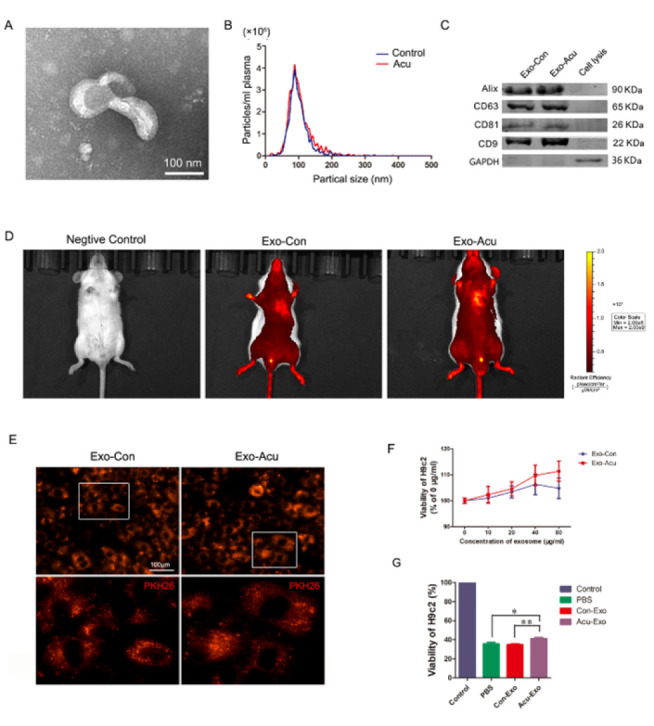
Characterization and functional validation of plasma exosomes derived from MI rats with and without acupuncture treatment

The plasma exosomes of MI rats were isolated with or without acupuncture (Exo-Con and Exo-Acu) at Neiguan (PC6) by using ultracentrifugation methods. Transmission electron microscopy (TEM) analysis of isolated pellets revealed a typical rounded bilayer-membrane structure with diameters of ~100 nm (Figure 4A). To determine the size of the pelleted structures, nanoparticle tracking analysis (NTA) was used, and the results showed that exosomes (Exo-Con and Exo-Acu) were approximately 90 nm in diameter, accounting for nearly 98% of the population, while the remaining particles ranged from 30 nm to 250 nm. Furthermore, the plasma concentration of Exo-Con was almost as much as that of Exo-Acu from the same amount of plasma (3.6×10^11^ versus 4.2×10^11^/ml, Figure 4B). Then, the Exo-Con and Exo-Acu samples underwent western blot analysis, revealing the presence of the exosome markers Alix, CD63, CD81, and CD9 (Figure 4C).

To clarify the tissue distribution of plasma exosomes, an IVIS® Lumina II small animal imaging system was used four hours after exosome administration via the tail vein. The signal produced by the DiI-labeled exosomes induced by acupuncture was primarily detected in the heart region of the MI mouse. The signal was also observed in the distal limbs and feet, perirhinal and perioral regions, external genitals, and tail. There was no noticeable difference in signal distribution between exosomes of the acupuncture and control groups. No signal was detected in the negative control mouse, which was injected with PBS via the tail vein (Figure 4D).

To confirm whether exosomes could be internalized through endocytosis, the Exo-Con and Exo-Acu were labeled with PKH26 dye and incubated separately with cultured H9c2 cells. Six hours later, an intense red fluorescence was observed in the H9c2 cells’ cytoplasm of the two groups, indicating the similar entry of Exo-Con and Exo-Acu into H9c2 cells (Figure 4E). 

To evaluate the influence of plasma exosomes on the viability of H9c2 cells, the CCK-8 assay was performed after incubating cells with different concentrations of exosomes (10, 20, 40, and 80 µg/ml) for 24 hr. Exo-Acu exhibited a concentration-dependent enhancement in the viability of H9c2 cells compared to Exo-Con, demonstrating superior efficacy. Given that the viability of H9c2 cells was enhanced at the highest concentration of 40 µg/ml Exo-Con, a similar concentration of Exo-Acu was employed for subsequent experiments. (Figure 4F).

For investigation of the cardioprotective effects of plasma exosomes, the H9c2 cells were pretreated with phosphate-buffered saline (PBS), Exo-Con, or Exo-Acu for 24 hr and subjected to H_2_O_2_ (0.4 mM) or PBS treatment for two hours. The evaluation of cellular viability was performed using the CCK-8 assay. The results showed an obvious increment of cell viability in the Exo-Acu-treated H9c2 cells. Notably, the viability of the H_2_O_2_-treated cells did not benefit from Exo-Con, similarly to that of the PBS-treated cells (Figure 4G). These results suggest that circulating exosomes could accumulate in the infarcted heart and be internalized into cardiomyocytes through cardiocytic endocytosis. However, only the exosomes induced by acupuncture at PC6 afforded cytoprotective effects to cardiomyocytes. 

### MiR-142-3p is a pivotal constituent present in circulating exosomes after acupuncture treatment

**Figure 5:**
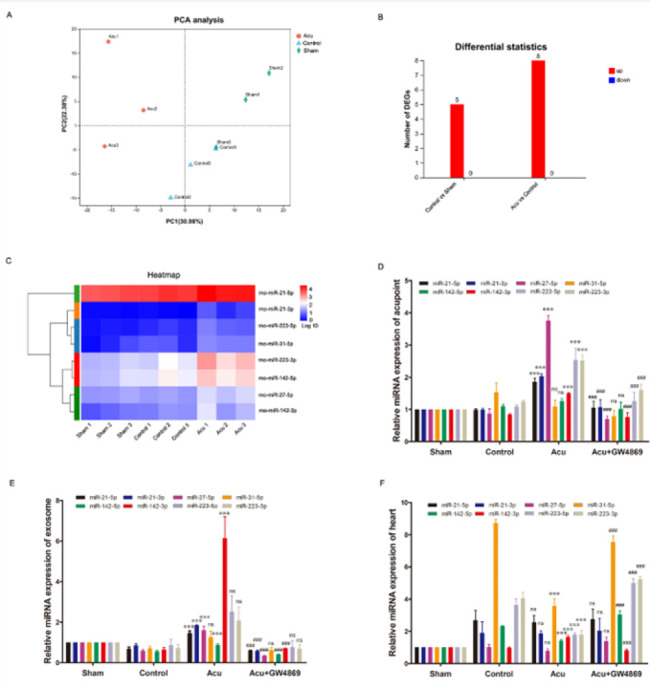
MiR-142-3p is a crucial exosomal miRNA induced by acupuncture in rats

The changes of miRNAs in the tissue of Neiguan (PC6) were tested by small RNA sequencing. First, to analyze the miRNA profiles among nine samples of PC6 acupoint tissues, principal component analysis (PCA) was conducted. The results showed that seven samples were categorized into sham, control, and acu groups; two samples from the sham and control groups (sham 3 and control 1) were more correlated than samples from the same group (Figure 5A). An analysis of differentially expressed genes (DEGs) was subsequently conducted, and eight differentially up-expressed miRNAs (miR-21-5p, miR-21-3p, miR-27-5p, miR-31-5p, miR-142-5p, miR-142-3p, miR-223-5p, and miR-223-3p; fold change >2.0; *P*<0.05; Figure 5B) were detected in the acupunctured PC6 acupoint tissue compared to the tissue without acupuncture. Third, a heatmap of the eight up-regulated miRNAs was generated; the results showed that the samples from the sham, control, and acu groups were clustered separately (Figure 5C).

Reinforcement of the small RNA sequencing data was achieved through real-time polymerase chain reaction (RT-PCR) analysis. Among eight up-expressed miRNAs, six (miR-21-5p, miR-21-3p, miR-27-5p, miR-142-3p, miR-223-5p, and miR-223-3p) were identifiably up-regulated in the Acu group compared with the control group (Figure 5D). The changes of these eight miRNAs in plasma exosomes were also investigated by RT-PCR analysis, and five of them (miR-21-5p, miR-21-3p, miR-27-5p, miR-142-5p, and miR-142-3p) were up-regulated in the Acu group compared with the control group (Figure 5E). Finally, the differential expression of these eight miRNAs in the heart was verified. The results demonstrated a unique up-regulation of miR-142-3p in the Acu group compared to the control group (Figure 5F). Interestingly, GW4869 reversed the miRNA changes in these tissues induced by acupuncture (Figures 5D-F). Collectively, these findings suggest that miR-142-3p, induced by acupuncture at Neiguan (PC6) and enriched in plasma exosomes as well as in the injured heart, plays an essential role in distal acupuncture-mediated remote cardioprotection.

### CFL2 is a target gene of miR-142-3p in acupuncture-induced cardioprotection

**Figure 6:**
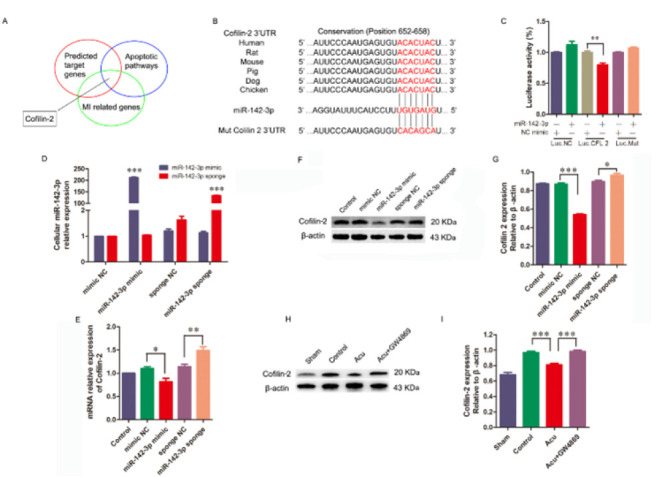
CFL2 is a target gene of miR-142-3p in cardioprotection of acupuncture in H9c2 cells

Bioinformatics-based analysis (miRanda, miRDB, and TargetScan) was employed to predict potential target genes of miR-142-3p in acupuncture-induced cardioprotection. The focus of this study lies on CFL2 (Figure 6A. Based on the bioinformatics analysis, miR-142-3p was predicted to bind the CFL2 3′-untranslated region (UTR), which contains a highly conserved binding site (652-658, Figure 6B). After the co-transfection of a luciferase reporter plasmid harboring either the wild type or mutant form of CFL2 3′-UTR, along with an miR-142-3p mimic or a scrambled miRNA into the 293 T cells, we evaluated the activity of luciferase. The results of the dual luciferase reporter assay indicated that introduction of the miR-142-3p mimic resulted in a specific suppression of CFL2 3′-UTR luciferase activity, whereas no inhibition in the mutant construct was observed (Figure 6C). Meanwhile, H9c2 cells were infected with a lentiviral vector to express a miRNA-142-3p mimic, a miR-142-3p sponge, or their respective controls, further to confirm the inhibition of CFL2 expression by miR-142-3p. The RT-PCR results showed a 100-fold increase in miRNAs induced by infection. The RT-PCR and western blot results revealed a significant decrease in CFL2 induced by the miR-142-3p mimic lentivirus and an increase in CFL2 induced by the miR-142-3p sponge lentivirus in H9c2 cells (Figure 6D-G). The *in vivo *expression of CFL2 was also assessed following acupuncture treatment, and the western blot analysis revealed a significant reduction in CFL2 levels in the Acu group compared to the control group. Conversely, an evident increase in CFL2 expression was observed in the Acu plus GW4869 group when compared to the Acu group (Figures 6H and I). The collective data suggest that CFL2 serves as a downstream target gene of miR-142-3p in the context of acupuncture-induced cardioprotection.

### Up-regulation of miR-142-3p attenuates the apoptotic effects induced by H2O2 in H9c2 cells

**Figure 7:**
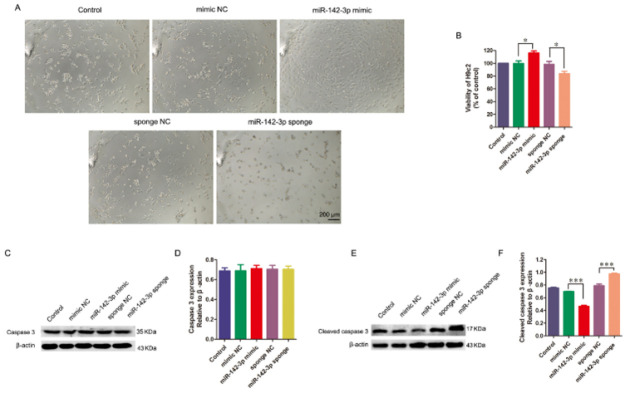
Overexpressing miR-142-3p retarded oxidative damage by anti-apoptosis in H9c2 cells

H9c2 cells overexpressing miR-142-3p mimic or sponge induced by lentivirus were incubated with 0.4 mM H_2_O_2 _for two hours to further investigate the functional impact of miR-142-3p on cardioprotection. Distinctive morphological changes were observed in the H9c2 cells of the control, mimic NC, and sponge NC groups, characterized by shrunken, rounded, and distorted cell shapes. These morphological changes were partly ameliorated in the miR-142-3p mimic group and worsened in the miR-142-3p sponge group (Figure 7A). The results of the CCK-8 assay showed that the miR-142-3p mimic increased the viability of H9c2 cells, whereas the miR-142-3p sponge decreased it (Figure 7B). To detect apoptosis of miR-142-3p overexpressing H9c2 cells treated by H_2_O_2_, caspase 3 and CC3 were assessed using western blot analysis. The results showed that miR-142-3p overexpression did not alter caspase-3 levels but reduced CC3 expression at the protein level in H9c2 cells, whereas the miR-142-3p sponge reversed this effect (Figures 7C-F). This data suggests that miR-142-3p has a protective effect on H_2_O_2_-induced injuries in H9c2 cells via anti-apoptotic mechanisms.

## Discussion

**Figure 8:**
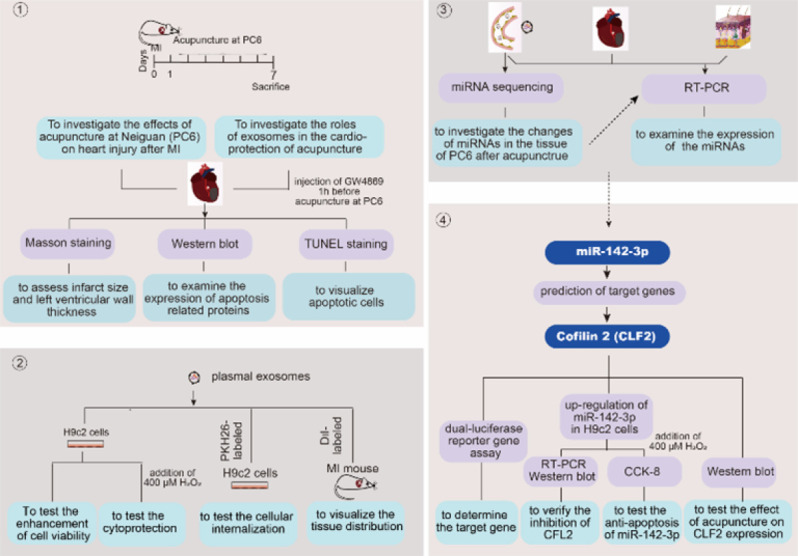
Schematic diagram illustrating the experimental design of the present study in rats and H9c2 cells

This study revealed two major findings. Firstly, acupuncture-induced circulating exosomes transfer cardioprotective signals to the injured heart, thereby conferring cardioprotective effects. Secondly, miR-142-3p is a major exosomal miRNA in inhibiting apoptotic signals by targeting CFL2. The aims, procedures, and methods were presented schematically in Figure 8.

The acupoint Neiguan (PC6) is the most commonly used in Chinese acupuncture theory for treating heart conditions. A wealth of evidence has shown the benefits of Neiguan (PC6) acupuncture in attenuating cardiac injury through multiple pathways, including the inhibition of apoptosis, alleviation of mitochondrial damage, and reduction in the degree of inflammation (12, 33, 34). In the present study, acupuncture at Neiguan (PC6) thickened the left ventricular wall in a rat model of MI and down-regulated the expression of caspase 3 and CC3. These results indicated that ischemic cardiocytes could benefit from remote stimulation via acupuncture and survive severe damage through the anti-apoptotic effects of acupuncture. However, the mechanism of distant acupuncture associated with protecting the injured heart remains unclear. 

Recent studies have discovered that circulating exosomes can transfer their signal molecules to recipient cells in the infarction area and enhance the heart’s function (15, 16, 35). Circulating exosomes represent a potentially central role of distance communication from Neiguan (PC6) to the injured heart. For confirmation of this assumption, exosome secretion of an MI rat was inhibited by consecutive intraperitoneal injections of GW4869 before each needling. The application of GW4869 significantly attenuated the beneficial impact of acupuncture on the infarcted heart, as evidenced by the noticeable reduction in protective effects observed through Masson’s trichrome staining, with a thinner left ventricular wall and fewer cardiocytes than the Acu group without GW4869 injection. Meanwhile, with the reduction in exosome secretion by GW4869, the acupuncture-induced inhibition of apoptosis in injured cells was attenuated, specifically manifesting as an increase in TUNEL-positive cells, upregulation of Bax, caspase 3, and CC3, and down-regulation of Bcl-2. Furthermore, H_2_O_2_-induced apoptosis was used to determine the cryoprotection of plasmatic exosomes on apoptosis in H9c2 cells. Consistent with the *in vivo* results, H9c2 cells incubated with exosomes from acupuncture rats exhibited higher viability than those incubated with exosomes from model rats. These results demonstrated the anti-apoptosis effects of exosomes induced by acupuncture at PC6 on myocardial tissue after MI. To further clarify the biodistribution of circulating exosomes, we harvested plasma exosomes of MI rats acupunctured at PC6 and non-acupoint separately, and labeled them with DiI. Four hours after exosome administration via the tail vein, most of the DiI-labeled exosomes accumulated in the heart region of the MI mouse. No noticeable difference in organ distribution was observed between Exo-Con and Exo-Acu. The similar entry of Exo-Con and Exo-Acu into H9c2 cells was also observed *in vitro*. These results implied that the cargo of circulating exosomes induced by acupuncture at PC6 might play pivotal roles in cardioprotection after MI.

MiRNAs have received more interest for their dramatic effects with minute quantities than proteins and mRNAs, which are also harbored in the exosome (36). A series of studies has clarified that circulating exosomes after acute MI carry myocardial miRNAs (such as miR-1, miR-208, miR-499, *etc*.) to regulate myocardial repair (37, 38). These studies suggest a novel therapeutic strategy for MI based on regulating the cargo of circulating exosomes (39). Based on the cardioprotective effects of acupuncture at PC6, we investigated changes in miRNAs in the PC6 tissue using small RNA sequencing. First, eight differently expressed miRNAs were found in the acupunctured tissue of PC6 compared with the non-acupunctured tissue of PC6. Among them, six miRNAs were verified by RT-PCR. To further explore whether these miRNAs were harbored in circular exosomes, RT-PCR was performed. The results showed that five miRNAs were differently expressed in the exosome, and the expression of miR-142-3p exhibited a significant increase among these miRNAs. Interestingly, miR-142-3p was found to be the unique up-regulating miRNA in the myocardial tissue of the Acu group. In addition, these miRNA changes induced by acupuncture were reversed by GW4869 injection. The results demonstrated that acupuncture affects the quality of circulating exosomes, and exosomes with miR-142-3p enrichment induced by acupuncture may exert a cardioprotective effect. The inhibitory effect of miR-142-3p on cardiomyocyte apoptosis following cardiac injury has been demonstrated to involve the activation of multiple signaling pathways. Su *et al*. reported that overexpression of miR-142-3p using agomiR improved cardiac function in a model of coronary microembolization-induced myocardial injury and attenuated the myocardial inflammatory response by targeting IRAK-1 (40). Similarly, Zhao *et al*. demonstrated that miR-142-3p exerted anti-apoptotic effects on cardiomyocytes in a murine ischemia-reperfusion (IR) model by inhibiting the TLR4/NF-κB axis (41).

Theoretically, a miRNA could target many different mRNAs. In the present study, the bioinformatics analysis revealed that miR-142-3p specifically targeted the mRNA of CFL2, as verified by the dual luciferase reporter system. CFL2 is the primary form of the actin-binding protein family in differentiated cardiocytes (42). CFL2 undergoes translocation to the mitochondria, inducing significantly enhanced apoptosis and playing a role in the response of myocardiocytes to oxidative stress (43, 44). A recent study indicated that CFL2 promotes apoptosis by translocating to the mitochondria, which leads to the release of cytochrome c and caspase activation (45). In this study, CFL2 expression was confirmed to be down-regulated by an miR-142-3p mimic and up-regulated by a miR-142-3p sponge in H9c2 cells. Moreover, acupuncture at Neiguan (PC6) decreased CFL2 expression in infarcted myocardium, and GW4869 injection reversed the suppression effect of acupuncture on CFL2. MiR-142-3p exhibits a pleiotropic role in CVDs, and it has been proven to be an important regulator for inhibiting inflammation, apoptosis, and autophagy in injured cardiomyocytes (40, 46). Through up- and down-expression of miR-142-3p, miR-142-3p was proven to protect H9c2 cells treated with H_2_O_2_ and down-regulate the expression of CC3. These findings indicate that miR-142-3p possesses anti-apoptotic effects, partly through down-regulation of CFL2 and the subsequent inhibition of caspase 3 in MI hearts.

There are some limitations in our study. Firstly, only Neiguan (PC6) was chosen in the present experiments to clarify the effect of acupuncture on miRNA expression, as this was the original purpose of the study. The impact of other acupoints with similar cardioprotective effects (such as ST36 and BL15) on exosomal miRNAs will be compared in further research. Secondly, we measured the changes in miRNAs in the tissue of Neiguan (PC6) induced by acupuncture; however, the cellular source of these miRNAs was not further analyzed because they have been proven to be expressed by many cells in the acupoint, such as keratinocytes, mast cells, lymphocytes, and others.

**Figure 9:**
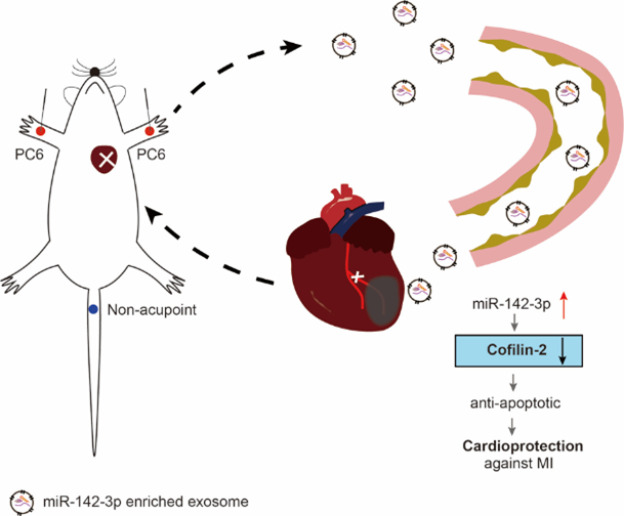
**. **Proposed mechanisms of acupuncture cardioprotection on the infarcted heart by inducing exosomes

In summary, plasma exosomes are distinctively ferrying carriers for cardioprotection of acupuncture. Exosomal miR-142-3p has been identified as a pivotal molecule possessing cardioprotective properties, effectively inhibiting apoptosis in cardiomyocytes. Moreover, the gene CFL2 was identified as a target of miR-142-3p. These findings presented a novel cardioprotective mechanism of remote acupuncture by inducing exosomal miR-142-3p, revealing the therapeutic potential of miR-142-3p in the prevention and repair of damage caused by ischemic heart disease (Figure 9).

A. photograph of the heart transverse section at the widest parts of the infarcted regions (bar=5 mm). The second and third rows show partial enlarged views of the infarcted regions in the upper row (bar=100 μm). B and C. Statistical analysis of the left ventricular wall thickness and scar size of the infarcted hearts from various groups of rats (*P<0.05; ***P<0.001 versus sham group; ^##^*P<*0.01 versus control group, n=6). D-F Western blot and quantified data for the expression of caspase 3 and cleaved caspase 3 (CC3) in infarcted cardiac tissue from various groups of rats. (*P<0.05, ***P<0.001 versus sham group; #*P<*0.05, ^##^*P<*0.01 versus control group, n=6)

A. Photographs of the heart transverse section at the widest parts of the infarcted regions (bar=5 mm). The second and third rows are the partial enlarged views of the infarcted regions in the first row (bar=100 μm). B and C. Statistical analysis of the LV wall thickness and scar size of the infarcted hearts from various groups of rats (***P<0.001 versus the sham group; ^##^*P<*0.01 versus control group; #P<0.05 versus acupuncture group, n=6)

A. TUNEL staining of the heart cross-section from various groups of rats. The third row shows a partial enlarged view of the infarcted regions in the box of the second row (bar=100 μm). B-E. western blot and quantified data for the expression of Bcl-2, BAX, caspase 3, and cleaved caspase 3 (CC3) in infarcted cardiac tissue from various groups of rats (*P<0.05, ***P<0.001 versus control group; ^#^*P<*0.05, ^###^*P<*0.001 versus acupuncture group, n=6)

A. Transmission electron micrograph of plasma exosomes. B. western blot assessing the presence of Alix, CD63, CD81, and CD9 in plasma exosomes. C. Nanoparticle trafficking analysis of the diameters and concentration of plasma exosomes. D. Biodistribution of DiI-labeled plasma exosomes. E. Uptake of PKH26-labeled plasma exosomes (red spots) by H9c2 cells. F. Cell viability of H9c2 incubated with different concentrations of exosomes. G. CCK-8 assay for detecting the viability of H9c2 cells incubated with 0.4mM H_2_O_2_ for two hours after pretreatment with Exo-Con or Exo-Acu (* *P<*0.05; ** *P<*0.001, n=6)

A. principal component analysis (PCA) analysis of 9 tissue samples of PC6. B. Differentially expressed genes (DEGs) analysis of sham, control, and Acu group. C. Expression heatmap showing the relative abundance of eight miRNAs in the tissue of Neiguan (PC6) treated with acupuncture (n=3). D. RT-PCR analysis of eight differentially expressed miRNAs in the tissue of Neiguan (PC6) from various groups of rats (n=6). E. RT-PCR analysis of eight differentially expressed miRNAs in exosomes from various groups of rats (n=6). F. RT-PCR analysis of eight differentially expressed miRNAs in the infarcted region of hearts from various groups of rats (n=6). ***P<0.001 versus control group; ^###^*P<*0.001 versus acupuncture group

A. Screening scheme for putative target genes that may contribute to the antiapoptotic effects of miR-342-5p. B. Luciferase reporter constructs containing wild-type or mutated 3′-UTR of rat CFL2 mRNA. The sequence in red indicates the predicted binding site for miR-142-3p, aligning with the miR-142-3p seed sequence. C. Relative luciferase activity normalized to negative control of miR-142-3p mimic (NC mimic) (CFL2, **P<0.01, n=3). D. RT-PCR analysis of miR-142-3p and miR-142-3p sponge expression in H9c2 infected by lentiviral vector expressing miRNA-142-3p mimic or miR-142-3p sponge (***P<0.01 versus negative control, n=3). E. RT-PCR analysis of CFL2 expression in H9c2 infected by lentiviral vector expressing miRNA-142-3p mimic or miR-142-3p sponge (*P<0.05; **P<0.01, n=3). F and G. western blot and quantified data for the expression of CFL2 in H9c2 cells infected by lentiviral vector expressing miRNA-142-3p mimic or miR-142-3p sponge (*P<0.05; ***P<0.001, n=3). H and I. western blot and quantified data for the expression of CFL2 in infarcted cardiac tissues from various groups of rats (***P<0.001, n=6)

H9c2 cells were infected with a lentiviral vector expressing miRNA-142-3p mimic or miR-142-3p sponge. A. Morphological change in H9c2 cells incubated with 0.4mM H_2_O_2_ for two hours. B. CCK-8 assay assessing the viability of H_2_O_2_-incubated H9c2 cells (*P<0.05, n=6). C-F. western blot and quantified data for the expression of caspase 3 and cleaved caspase 3 (CC3) in H_2_O_2_-incubated H9c2 cells (***P<0.001, n=6)

Plasma exosomes induced by acupuncture offer cardioprotection through anti-apoptosis, and exosomal miR-142-3p is a pivotal molecule that suppresses Cofilin 2 expression in the infarcted heart and inhibits apoptosis of the infarcted myocardium

## Conclusion

Our results suggest that plasma exosomes transfer cardio-protective signals from acupuncture to the injured heart, conferring cardioprotective effects. Furthermore, miR-142-3p emerges as a prominent exosomal miRNA in the inhibition of myocardial apoptosis by targeting CFL2.

## Declaration

We have not used any AI tools or technologies to prepare this manuscript.
